# Impact of prior robotic surgical expertise on the results of Hugo RAS radical prostatectomy: a propensity score-matched comparison between Da Vinci-expert and non-Da Vinci-expert surgeons

**DOI:** 10.1007/s00345-025-05608-2

**Published:** 2025-04-20

**Authors:** Filippo Gavi, Maria Chiara Sighinolfi, Daniele Fettucciari, Cristina Carerj, Filippo Marino, Enrico Panio, Pierluigi Russo, Nazario Foschi, Riccardo Bientinesi, Carlo Gandi, Giuseppe Palermo, Angelo Totaro, Emilio Sacco, Bernardo Rocco

**Affiliations:** 1 Fondazione Policlinico Universitario Agostino Gemelli IRCCS, Rome, Italy; 2 Humanitas Gavazzeni, Bergamo, Italy; 3 Ospedale Isola Tiberina - Gemelli Isola, Rome, Italy

**Keywords:** Prostate cancer, Robotic surgery, Hugo RAS, Da Vinci system, Robotic surgeon

## Abstract

**Background:**

Hugo RAS is a novel robotic platform gaining global adoption. Most reported outcomes come from centers with prior Da Vinci experience, with limited data from robotic-naïve settings or comparisons based on prior robotic expertise.

**Objective:**

To compare outcomes of Hugo RAS robot-assisted radical prostatectomy (RARP) performed by Da Vinci-experienced (DVE) versus non-Da Vinci-experienced (NDVE) surgeons. Design, Setting, and Participants: Prospective data from patients undergoing Hugo-RARP (July 2022–November 2024) were analyzed. Patients were grouped based on whether their surgeon had prior Da Vinci experience. None had prior Hugo-RAS experience. Outcome Measurements and Statistical Analysis: Primary outcomes were positive surgical margin (PSM) and complication rates. Secondary outcomes included operative time (OT), estimated blood loss (EBL), length of stay (LOS), continence, and potency. Propensity score matching adjusted for baseline differences.

**Results and limitations:**

After matching, 117 patients per group were analyzed. PSM rates (17% vs. 21%; *p* = 0.40) and complications (*p* = 0.63) were similar. DVE surgeons had shorter OT (179 vs. 206 min; *p* < 0.001) and lower EBL (127 vs. 161 ml; *p* = 0.008). LOS did not differ (*p* = 0.84), and 12-month functional and oncological outcomes were comparable. Limitations include the non-randomized, single-center design.

**Conclusions:**

Hugo RAS enables safe and effective RARP with comparable outcomes regardless of prior robotic experience. Prior Da Vinci experience, however, improves intraoperative efficiency.

**Supplementary Information:**

The online version contains supplementary material available at 10.1007/s00345-025-05608-2.

## Introduction

Robot assisted surgical systems have experienced notable improvement and worldwide adoption in the last 20 years. However, access to robotic surgery has been limited by the availability of robotic systems and concerns about cost-effectiveness [1, 2]. Several novel robotic platform are emerging and are still evolving [3]. The novel robotic platforms offer opportunity for technological improvement and cost reduction [4, 5]. Hugo robot-assisted surgery (RAS) (Medtronic, Minneapolis, MN, USA) was introduced in the market in 2022 after receiving European CE approval for urological procedures in adults. Several studies and metanalysis have described the feasibility of the RARP with Hugo RAS (Hugo-RARP) showing comparable results with Da Vinci system by Intuitive [6–8], but high quality and long term follow up data regarding clinical and perioperative outcomes are lacking [5, 9, 10]. Furthermore, most of the published experience on Hugo RAS derive from referral centers with a consolidated experience with the Da Vinci. The introduction of Hugo RAS in a robotic naïve setting is, to now, a less reported topic and the impact of a prior Da Vinci experience is yet unknown.

Therefore, we aimed to determine whether surgeons’ prior experience with the da Vinci robot impacts perioperative and oncological outcomes of radical prostatectomy performed with the Hugo RAS system.

## Materials and methods

This is a prospective nonrandomized study on radical prostatectomies performed at the Urologic Department of Fondazione Policlinico Universitario Agostino Gemelli IRCCS. Since July 2022, all radical prostatectomies were performed with the Hugo RAS. Data collection obtained IRB approval (approval number: 5119/2022) and all patients signed an informed consent to the procedure. The aim of the study is to compare outcomes of RARP performed by Da Vinci expert surgeons (DVE) (two surgeons) to those of patients undergoing surgery from Non-Da Vinci expert surgeons (NDVE) (three surgeons). Data of patients from our prospectively maintained institutional database were used to create two cohorts: patients operated on by two expert surgeons with more than 100 cases with Da Vinci and three surgeons with non-Da Vinci experience. The expert surgeons performed > 100 cases with the Da Vinci Xi before January 2022. The surgeons in their learning curve had no experience in robot assisted prostatectomy with Da Vinci nor laparoscopy. All surgeons involved in our study received a specific dry- and wet-lab training sessions on the Hugo RAS docking system and console controls at ORSI Academy. During the first 20 cases the expert surgeons remained in the OR without sitting in the console. Surgeons were assigned cases based on routine clinical practice. Data collection was achieved up to 24 months after surgery by phone calls or visits, all performed by medical personnel not directly involved in surgery.

### Primary endpoint

The primary endpoint is the evaluation of the safety of Hugo-RARP performed by DVE and NDVE surgeons. Outcome measures were positive surgical margin (PSM) rate and complication rate, classified according to Clavien Dindo system [11]. PSM was defined as the presence of tumor cells at the inked resection margin in the surgical specimen.

### Secondary endpoint

Secondary endpoints were intra- and post-operative variables including operatory time (OT, minutes); estimated blood loss (EBL, mL); length of stay (LOS; days). Biochemical recurrence (BCR), continence and potency were recorded as secondary endpoints. Urinary continence was outlined according to a “no pad/safety pad” definition. Potency, described as the ability to achieve erections adequate for intercourse, was evaluated in patients under 65 who had undergone complete nerve-sparing dissection and allowed the use of PDE-5 inhibitors.

### Surgical technique

All procedures were conducted using the Hugo RAS, adhering to the Montsouris technique [12]. The surgical approach and system setup for performing RARP with the Hugo RAS have been previously detailed and published [13]. Lymph node dissection was carried out selectively based on the preoperative risk of nodal invasion, as calculated by the 2018 Briganti nomogram [14].

### Statistical analysis and propensity score matching

Both the Shapiro-Wilk test and the graphical assessment were adopted for assessing data distribution. The mean and standard deviation (SD) were calculated for continuous variables. In the case of normal distribution, to compare the means of two or more continuous variables, the Student t-test was utilized whereas the Mann-Whitney U test was deemed suitable for the nonparametric variables. For categorical variables, the Pearson χ2 test was applied. Propensity score matching was conducted based on preoperative variables, including age, Charlson Comorbidity Index (CCI), history of abdominal or BPH surgery and total prostate-specific antigen (PSA) levels (ng/ml), using caliper widths set at 0.2 of the SD. Matching success was confirmed when the absolute standardized mean difference after matching was < 0.20. All statistical analyses were conducted in STATA 18 (Statistical Software: Release 18. Stata Corp LLC), with statistical significance set at a two-tailed *p* < 0.05.

## Results

A total of 272 Hugo-RARP procedures were conducted during the study period, with 121 performed by DVE and 151 by NDVE. Following propensity score matching, two balanced groups of 117 patients each were formed. All covariates achieved balance after matching, except for the CCI. No significant differences were found between groups in terms of previous surgeries, prostate volume, or oncological clinical characteristics. A summary of data and balance information before and after matching is provided in Table 1 and in the supplementary materials.

**Table 1:** Baseline characteristics of the two groups before and after matching

Variable	Before matching				After matching			
	Non Da Vinci Expert (*n* = 151)	Da Vinci Expert (*n* = 121)	*P* Value	SMD	Non Da Vinci Expert (*n* = 117)	Da Vinci Expert (*n* = 117)	*P* Value	SMD
Age (mean ± SD)	66 ± 7	65 ± 8	0.80	0.23	66 ± 6	66 ± 7	0.88	0.13
PSA (ng/ml; mean ± SD)	9.4 ± 7	9.9 ± 7	0.53	-0.54	8.9 ± 7	10.0 ± 7	0.22	-1.10
CCI score, n (%)			0.10				0.04	
< 3	68 (45)	75 (62)			56 (48)	73 (62)		
4–6	74 (49)	44 (36)			59 (49)	42 (36)		
7–9	6 (4)	2 (2)			2 (2)	2 (2)		
cT, n (%)			0.65				0.58	
T1c	60 (40)	56 (43)			54 (63)	55 (59)		
T2a	26 (17)	18 (15)			24 (28)	17 (19)		
T2b	27 (22)	28 (23)			20 (23)	26 (30)		
T2c	18 (12)	13 (11)			15 (17)	13 (15)		
> T2	9 (6)	6 (5)			4 (3)	6 (7)		
Gleason score at biopsy, n (%)			0.09				0.30	
6	36 (23)	38 (31)			34 (29)	37 (32)		
7	95 (63)	61 (50)			70 (60)	60 (51)		
8–10	20 (13)	22 (18)			13 (11)	20 (17)		
D’Amico risk group, n (%)			0.16				0.16	
Low	26 (17)	24 (20)			23 (20)	24 (21)		
Intermediate	87 (58)	56 (46)			67 (57)	54 (46)		
High	38 (25)	41 (34)			27 (23)	39 (33)		
Previous abdominal surgery (%)	44 (30)	35 (29)	0.96		33 (28)	34 (29)	0.88	
Previous BPH surgery, n (%)	7 (5)	6 (5)	0.90		4 (3)	6 (5)	0.51	
Prostate volume (ml; mean ± SD)	50 ± 19	50 ± 26	0.75	-0.86	50 ± 20	49 ± 26	0.80	0.76

BPH = benign prostatic hyperplasia; CCI = Charlson Comorbidity Index; PSA = prostate-specific antigen; SD = standard deviation; SMD = standardized mean difference. ±

Overall, PSM rate did not differ between the groups (17% vs. 21%; *p* = 0.40). Clavien-Dindo complications were rare and similar across both groups (*p* = 0.63). DVE surgeons had shorter OT (mean 206 min, SD = 68) compared to NDVE (mean 179 min, SD = 40; *p* = 0.0004); however, nerve-sparing surgery was more frequently performed bilaterally in the DVE group (21% vs. 32%; *p* = 0.004). EBL was higher in the NDVE group (mean 161 ml, SD = 112 vs. mean 127 ml, SD = 87; *p* = 0.008). Pathological findings revealed a worse pT stage in the DVE group, with 18% pT3a and 9% pT3b-4 compared to 6% and 11%, respectively, in the NDVE group (*p* = 0.01). However, no differences were observed in nodal stage (*p* = 0.75) or pathological ISUP grade (*p* = 0.23). One-year BCR was not different between groups (11% in the NDVE and 11% in the DVE group, *p* = 0.86). Intra- and postoperative outcomes are summarized in Table 2.

**Table 2:** Intra– and postoperative outcomes in matched populations

Variable	Non Da Vinci Expert (*n* = 117)	Da Vinci Expert (*n* = 117)	*P* value
Positive surgical margins, n (%)			
Overall	20 (17)	25 (21)	0.40
In > pT2	6 (30)	13 (41)	0.38
BCR at 12 months	11 (11)	13 (11)	0.86
pT, n (%)			0.01
T2	97 (83)	86 (73)	
T3a	7 (6)	21 (18)	
T3b-4	13 (11)	10 (9)	
Surgical time (min; mean ± SD)	206 ± 68	179 ± 40	0.0004
LND, n (%)	25 (21)	27 (23)	0.75
Nerve sparing			0.004
Full	24 (21)	38 (32)	
Partial	23 (19)	29 (35)	
Blood loss (ml; mean ± SD)	161 (112)	127 (87)	0.008
Clavien-Dindo complications, n (%)			0.63
1–2	10 (7)	4 (3)	
3a	0	1 (1)	
3b	1 (1)	1 (1)	
4	2 (2)	1 (1)	
Catheter removal (POD; mean ± SD)	10 ± 4	10 ± 6	0.84
LOS (day; mean ± SD)	4 ± 2	4 ± 1	0.84
Hospital readmission, n (%)	6 (5)	1 (1)	0.054
Follow-up (mo; mean ± SD)	9 ± 5	15 ± 9	< 0.001
pN, n (%)			0.76
N+	31 (26)	23 (28)	
Pathologic ISUP grade, n (%)			0.23
1	17 (14)	19 (16)	
2	63 (62)	65 (56)	
3	24 (21)	23 (20)	
4	0	4 (3.4)	
5	3 (3)	6 (5)	
Urinary continence			
At 90 days	91 (88)	93 (85)	0.64
At 12 months	63 (91)	82 (94)	0.83
Potency (< 65 year and full nerve sparing)	7 (47)^a^	25 (70)^b^	0.12

BCR = Biochemical recurrence; ISUP = International Society of Urologic Pathologists; LND = lymph node dissection; POD = postoperative day; SD = standard deviation; ^a^ Fifteen patients; ^b^ Thirty-six patients

## Discussion

The effective use of Hugo RAS for radical prostatectomy has been widely reported in the literature [7–9], with outcomes similar to those obtained with the Da Vinci. However, there is lack of evidence about the introduction of Hugo RAS in a robotic naïve setting.

From the current study, there is no difference in PSM and complication rate between prior Da Vinci surgeons and robot-naive ones, both approaching Hugo RAS technology for RARP. Longer OT and higher EBL were evident in the NDVE group but did not reflect into significantly higher morbidity of surgery. Docking time was not considered an essential variable, but OT could be longer than usual with HUGO RAS, as reported in a recent meta-analysis [15]. However, in our experience, the docking time significantly shortens over successive surgeries with HUGO RAS. It is worth noting that fewer nerve-sparing procedures were performed in the NDVE cohort, which could have made the difference in EBL even more significant. No differences were observed in BCR rate at 12 months. Continence and potency outcomes were similar. However, since potency data were available for only a small cohort, these results should be interpreted with caution.

To the best of our knowledge, this is the first study addressing the issue of a safe and effective Hugo RAS introduction within a robotic-naïve cohort of surgeons. Historically, longer robotic experience has been associated with less complications [16] and better functional outcomes [17]. Some studies showed that surgeons’ experience was not associated with higher risk of positive surgical margins [18, 19]. However, in a recent multicentric study Bravi et al. [20] demonstrated that increasing experience was associated with lower risk of positive surgical margins decreasing until the 500th case, therefore the issue is still controversial. Similarly, in other type of surgeries surgical experience was correlated with better results [21, 22]. Similarly, urinary continence recovery and other post-operative endpoints are seemingly associated with surgical expertise [17, 18, 23–26]; however, there is no consensus on duration of the learning curve for RARP.

For new technologies and new robotic platforms, the concept of learning curve is essential. The first patients treated could experience worse outcomes because of a less experienced surgeons, less effective robotic platform and shorter experience of mentors with the new system [27]. For centers adopting robotic surgery, structured training programs, simulation-based practice, and mentorship have been proved to shorten the learning curve. Several studies have been published comparing outcomes between the Da Vinci by Intuitive and the Hugo RAS by Medtronic showing comparable results [6, 28, 29] and in our recent metanalysis [8] we also confirmed comparable surgical and clinical data. In all the studies dealing with this new robotic platform, no data were reported on the impact of surgeons’ prior robotic expertise.

A prior study addressed the topic of skill transfer across platforms in a pre-clinical setting: Sighinolfi et al. [30] analyzed which factors may impact basic surgical skills at Hugo RAS simulator and found that among different variables– such as age, videogame use etc.– the prior console expertise is the only one impacting on skill performance and time of exercises. This is seemingly in contrast with the results of the current study; however, skills at the simulators are different from surgical achievements in the real-life practice and are not guarantee of best clinical outcomes.

The current study is not exempt from limitations. First, the non-randomized fashion limits its strength; we tried to overcome this limit with the use of the propensity score matching that aiming to reduce cofounding biases among the two groups. Second, monocentric study may be considered a limitation as well. The difference in pT3a rates may impact oncological outcomes, but the small sample limits conclusions. Despite PSM balancing, residual confounding from unmeasured variables remains a limitation.

However, it should be recognized that this is the first study assessing the clinical impact of prior console experience on surgical outcomes of RARP. We used the data from the largest dataset on Hugo RARP available to date (> 300 cases) and we included the results from different surgeons to overcome potential bias. Moreover, all prostate cancer patients were suddenly shifted to a Hugo-RARP surgery independently of the risk group, thus reflecting a realistic clinical practice. Further studies collecting data from different institutions are awaited to confirm - or not - our outcomes.

## Conclusion

No significant differences were observed in positive surgical margin and complication rates, between experienced robotic surgeons with the Da Vinci system and novice robotic surgeons performing radical prostatectomy with the HUGO RAS system. However, novice robotic surgeons required more time to perform RARP and experienced higher estimated blood loss. Further studies are required to provide more insights on the topic.

## Electronic supplementary material

Below is the link to the electronic supplementary material.

Supplementary Material 1

## Data Availability

No datasets were generated or analysed during the current study.
