# Supplementary material for: Impact of prior robotic surgical expertise on the results of Hugo RAS radical prostatectomy: a propensity score-matched comparison between Da Vinci-expert and non-Da Vinci-expert surgeons
Source: World J Urol. 2025 Apr 20;43(1):236. doi: 10.1007/s00345-025-05608-2 (PMC12009775; doi:10.1007/s00345-025-05608-2)
Supplement: Supplementary file 1 — Supplementary Material 1 [file 345_2025_5608_MOESM1_ESM.docx]

Supplementary materials.

Graph 1. Propensity score graph Da Vinci vs. Non-Da Vinci experts.

| Intra – and postoperative outcomes in unmatched populations. | | | |
| --- | --- | --- | --- |
| Variable | Non Da Vinci Expert (n = 151) | Da Vinci Expert  (n = 121) | P value |
| Positive surgical margins, n (%) |  |  |  |
| Overall | (18) | 25 (22) | 0.50 |
| In > pT2 |  |  | 0.38 |
| BCR at 12 months | (9) | (11) | 0.63 |
| pT, n (%) |  |  | 0.051 |
| T2 | 111 (73) | 87 (72) |  |
| T3a | 17 (11) | 24 (18) |  |
| T3b-4 | 23 (15) | 10 (9) |  |
| Surgical time (min; mean ± SD) | 207 ± 65 | 179 ± 40 | 0.0001 |
| LND, n (%) | 35 (23) | 28 (23) | 0.75 |
| Nerve sparing |  | | 0.002 |
| Full | 32 (21) | 34 (28) |  |
| Partial | 27 (18) | 38 (31) |  |
| Blood loss (ml; mean ± SD) | 161 ± 120 | 127 ± 85 | 0.005 |
| Clavien-Dindo complications, n (%) |  |  | 0.57 |
| 1-2 | 11 (7) | 4 (3) |  |
| 3a | 1 (1) | 1 (1) |  |
| 3b | 1 (1) | 1 (1) |  |
| 4 | 3 (2) | 1 (1) |  |
| Catheter removal (POD; mean ± SD) | 10 ± 4 | 10 ± 6 | 0.84 |
| LOS (day; mean ± SD) | 4 ± 2 | 4 ± 1 | 0.84 |
| Hospital readmission, n (%) | 8 (5) | 2 (2) | 0.051 |
| Follow-up (mo; mean ± SD) | 9 ± 5 | 15 ± 9 | < 0.001 |
| pN, n (%) |  |  | 0.66 |
| N+ | 31 (26) | 33 (28) |  |
| Pathologic ISUP grade, n (%) |  |  | 0.43 |
| 1 | 21 (14) | 19 (16) |  |
| 2 | 93 (62) | 68 (56) |  |
| 3 | 31 (21) | 24 (20) |  |
| 4 | 0 | 5 (4) |  |
| 5 | 4 (3) | 6 (5) |  |
| BCR = Biochemical recurrence; ISUP = International Society of Urologic Pathologists; LND = lymph node dissection; POD = postoperative day; SD = standard deviation; | | | |
